# Action Experience and Action Discovery in Medicated Individuals with Parkinson’s Disease

**DOI:** 10.3389/fnhum.2016.00427

**Published:** 2016-08-25

**Authors:** Jeffery G. Bednark, John N. J. Reynolds, Tom Stafford, Peter Redgrave, Elizabeth A. Franz

**Affiliations:** ^1^Queensland Brain Institute, The University of QueenslandBrisbane St Lucia, QLD, Australia; ^2^Department of Anatomy, Otago School of Medical Sciences, and The Brain Health Research Centre, University of OtagoDunedin, New Zealand; ^3^Department of Psychology, University of SheffieldSheffield, UK; ^4^Department of Psychology and fMRIotago, University of OtagoDunedin, New Zealand

**Keywords:** Parkinson’s disease, action discovery, ERPs, agency

## Abstract

Parkinson’s disease (PD) is a neurodegenerative disorder that markedly affects voluntary action. While regular dopamine treatment can help restore motor function, dopamine also influences cognitive portions of the action system. Previous studies have demonstrated that dopamine medication boosts action-effect associations, which are crucial for the discovery of new voluntary actions. In the present study, we investigated whether neural processes involved in the discovery of new actions are altered in PD participants on regular dopamine treatment, compared to healthy age-matched controls. We recorded brain electroencephalography (EEG) activity while PD patients and age-matched controls performed action discovery (AD) and action control tasks. We found that the novelty P3, a component normally present when there is uncertainty about the occurrence of the sensory effect, was enhanced in PD patients. However, AD was maintained in PD patients, and the novelty P3 demonstrated normal learning-related reductions. Crucially, we found that in PD patients the causal association between an action and its resulting sensory outcome did not modulate the amplitude of the feedback correct-related positivity (fCRP), an EEG component sensitive to the association between an action and its resulting effect. Collectively, these preliminary results suggest that the formation of long-term action-outcome representations may be maintained in PD patients on regular dopamine treatment, but the initial experience of action-effect association may be affected.

## Introduction

Parkinson’s disease (PD) is a neurodegenerative disorder caused by the progressive loss of dopamine neurons (Agid and Blin, 1987), which affects both motor and non-motor function. While dopamine medication (usually in the form of levodopa: L-dopa, a precursor of brain dopamine) is commonly used to restore motor function, exogenous elevation of dopamine has been linked to changes in dopamine-related signaling in the basal ganglia (e.g., Gotham et al., 1988; Cools et al., 2001, 2010; Frank et al., 2004; O’Reilly and Frank, 2006; Moustafa et al., 2008). Individuals with PD who are *ON* dopamine medication are shown to have increased sensitivity to reward feedback (Frank et al., 2004), exaggerated working memory updating (Moustafa et al., 2008), and enhanced action-outcome binding (Moore et al., 2010).

Crucially, the dopaminergic-system has a significant modulatory role in learning (Reynolds et al., 2001), and likely shapes the discovery of novel action-sensory outcome associations that make up adaptive behavior (Redgrave and Gurney, 2006; Redgrave et al., 2008, 2011; Franz, 2012). Based on the temporal dynamics of dopamine activity, it has been recently proposed that the dopamine-system provides both fast responses to salient events and slow responses that are only executed after the information has been highly processed (Joshua et al., 2009). This allows the dopamine-system to participate in both the monitoring of salient stimuli and the evaluation of information for modifying future behavior. These dynamic signals, however, may be affected in dopamine-medicated individuals with PD leading to changes in the monitoring and evaluation of behaviorally relevant stimuli.

Detecting the occurrence of salient external events and evaluating the cause of salient events is essential for learning and maintaining adaptive behavior (Redgrave and Gurney, 2006; Redgrave et al., 2008). Using event-related potentials (ERPs), derived from electroencephalography (EEG), we have previously shown that the discovery of novel action-outcome associations is dependent on an interaction between salience monitoring and evaluation of novel sensory information with regard to the preceding movement (Bednark et al., 2013). Two key ERPs were demonstrated to play a role: the feedback correct-related positivity (fCRP), and the novelty P3.

The fCRP, along with other reward-related potentials, is commonly associated with the monitoring of salient, positive and reward-related outcomes (Holroyd et al., 2008, 2011). Related to action, previous work has shown that the fCRP response to salient outcomes is enhanced when participants have a sense of control over the salient outcome (Li et al., 2011; Bednark et al., 2013; Bednark and Franz, 2014). Conversely, the novelty P3 is thought to reflect the engagement of evaluative processes necessary for learning when a novel stimulus is encountered (Friedman et al., 2001; Jongsma et al., 2006; Sailer et al., 2010). We have shown that the learning of novel actions directly modulates the novelty P3, with the amplitude of the novelty P3 in response to the behaviorally relevant outcome decreasing as task performance improves and participants acquired a novel action (Bednark et al., 2013). Importantly, both of these brain potentials have been associated with dopamine activity (de Bruijn et al., 2005; Poceta et al., 2006; Polich and Criado, 2006; Potts et al., 2006; Holroyd et al., 2008) and may separately reflect dopamine-related salience monitoring and evaluative processes.

In the present study, we use these brain potentials to investigate whether the dynamics of the dopamine-system, associated with the discovery of new actions, are altered in individuals with PD while *ON* regular dopamine treatment (forms of L-dopa), compared to healthy age-matched controls. To test this, medicated individuals with PD and age-matched controls performed action discovery (AD) and action control tasks similar to our previous study (Bednark et al., 2013). Given that dopamine is implicated in mediating both the fCRP and the novelty P3, we expect that both potentials will be enhanced in PD patients compared to controls. However, dopamine-medication boosting of action-effect associations is hypothesized to alter AD.

## Materials and Methods

### Participants

Eight patients with mild to moderate PD were recruited from the participant pool of the Action, Brain, and Cognition Laboratory at Otago, a program that works in close association with the Otago Parkinson’s Society, Dunedin, New Zealand (see Table 1 for patient details). The inclusion criteria were: no known dementia (Mini-Mental State Exam score ≥27), no current depressive symptoms (Geriatric Depression Scale score ≤5), and currently on dopamine medication.

**Table 1:** **Demographic, pathology, and drug details in Parkinson’s disease (PD) patients and controls**.

Patient ID	Gender	Age	UPDRS (motor)	Duration of disease	GDS	MMSE	Medication (mg/day)
1	F	49	17	8	1	30	Levodopa (150), benserazide (37.5), orphenadrine (50)
2	F	77	5	8	0	26	Levodopa (100), benserazide (28.5), lisuride (0.2), orphenadrine (50)
3	M	72	8	7	2	30	Levodopa (400), carbidopa (100), benztropine (1)
4	F	62	44	12	1	29	Levodopa (96), benserazide (24), ropinirole (8)
5	F	59	18	3	2	29	Levodopa (700), carbidopa (175)
6	F	66	49	9	2	28	Levodopa (400), carbidopa (100)
7	F	68	11	2	2	28	Levodopa (800), carbidopa (200), ropinirole (4.5)
8	M	69	42	9	4	29	Levodopa (1950), carbidopa (487.5), entacapone (600)
PD Mean (SD)	2M and 7F	65.25 (8.65)	24.25 (17.81)	7.25 (3.28)	1.75 (1.17)	28.63 (1.30)	
AMC Mean (SD)	2M and 7F	64.88 (9.25)	-	-	1.13 (1.46)	29.63 (0.52)	

Eight age- and sex-matched healthy controls were recruited from a University of Otago database of older adults participants (Table 1). Inclusion criteria were: no known neurological or psychiatric illnesses, no known dementia (MMSE score ≥27), and no current depressive symptoms (GDS score ≤5). The Lower South Otago Regional Ethics Committee approved all procedures. All participants volunteered and gave their fully informed written consent.

### Experimental Procedure

Prior to the experimental session, patients and controls were interviewed. During the interview, current medical history was obtained, including current PD treatment medication for patients. Neuropsychological tests were also administered to screen for dementia and depression. The motor portion of the UPDRS was administered to patients.

#### Tasks

For the AD and two action control tasks, participants were informed that their goal was to cause a green circle to appear in the center of the screen by moving the cursor on the screen using a tracking-ball mouse. They were further instructed to look at the fixation cross throughout the trials.

**Figure 1:**
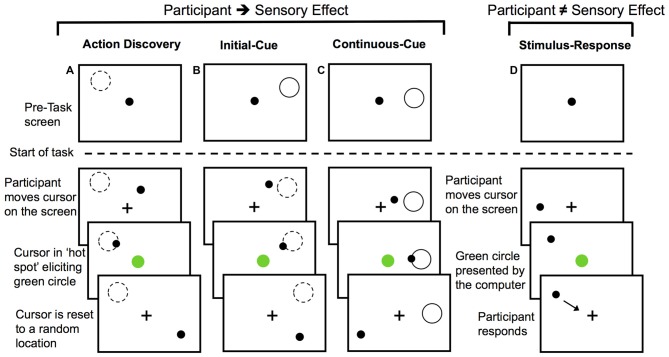
**Diagram of the experimental tasks.** For action discovery (AD) task **(A)**, the initial-cue (IC) task **(B)**, and the continuous-cue (cc) task **(C)** the sensory effect (green circle) was presented when participants moved the cursor into the “hot spot”. In the action control tasks **(B,C)**, the pre-task screen provided information about the location of the “hot spot”. After each presentation of the sensory effect the cursor was reset to a new location. Participants attempted to elicit the sensory effect after each cursor reset. Dashed-line circle indicates the location of the “hot spot” when it was not visible to the participant and the green circle represents the sensory effect. In the stimulus-response (SR) task **(D)** participants made voluntary movements, however, the presentation of the sensory effect was unrelated to their movements. Presentation of the sensory effect was computer-controlled. Participants responded to the green circle by moving the cursor to the fixation cross and then continued to make voluntary movements around the screen.

For the AD task, participants were not given any specific instructions about how to elicit the green circle, but they were told that they would learn how to do so over the course of the task. A diagram of the AD task can be seen in Figure 1A. The green circle was elicited by the participants’ movements when the cursor was moved into a specific location or “hot spot”. The location of the “hot spot” remained the same within a block of 30 trials or 30 presentations of the green circle, but each block had a different location for the “hot spot”. The cursor was re-located to a new random starting point after each presentation of the green circle. This was done so that the final position, and not the initial position of the movement, was the critical determinant of eliciting the presentation of the green circle. Participants had to then re-locate this location for each trial.

During the AD task, the monitoring and evaluation of the sensory effect was required to identify the movement(s) eliciting the sensory outcome. Over the course of the AD task, participants learned a set of efficient rules that guided action selection or a movement heuristic. This AD task was compared to two non-learning goal-directed motor tasks to investigate how the monitoring and evaluation of sensory outcomes may differ during AD. As a final control task, we used a stimulus-response (SR) task to investigate the monitoring of behaviorally relevant sensory effects that were unrelated to the preceding voluntary movement.

In the initial cue (IC) action-control task, location of the “hot spot” for eliciting the green circle was cued at the start of the block with a gray outline of a circle (Figure 1B). Participants were instructed that moving the cursor into this screen location would elicit the green circle, but that the gray outline circle would not be present when they performed the task. The cue was then removed and participants began this IC task. This IC provided participants with sensory information for performing the necessary actions for achieving the task goal prior to the start of the task, thereby serving as a control for learning novel actions during the AD task.

The continuous cue (CC) action-control task served as a control for any memory processes or uncertainty (particularly with regards to the novelty P3) associated with remembering the cued location in the IC task or the learnt location in the AD task. At the start of the CC task, participants were instructed that moving the cursor into the location defined by a gray outline of a circle on the screen would elicit the green circle (Figure 1C). For action-outcome task (Figures 1A–C) the cursor was re-positioned to a different location on the screen following each presentation of the green circle.

The final control task was a SR task (Figure 1D). For the SR task, participants were informed that their movements did not elicit the sensory effect. Rather, they were instructed to move the cursor around the screen (so participants were still making voluntary movements) and respond to the presentation of the green circle by moving the cursor to the fixation cross (so the green circle was still salient) and then resume moving the cursor around the screen. To control for the reduction in the inter-stimulus interval, which is known to affect P3 amplitude (e.g., Gonsalvez et al., 2007) that is observed with learning in the AD task, we controlled the timing of green circle presentation. The timing of the green circle in each block of the SR task was computer-controlled to have the same inter-stimulus interval as the corresponding block of the movement-learning task performed by the same participant (thereby using yoked timing conditions). As a result, any variation or decrease in the timing interval of the sensory effects in the movement-learning task would be directly controlled for by the SR task.

The behavioral tasks that were administered during the experimental session were previously conducted in normal undergraduate participants (Bednark et al., 2013). The theoretical importance of our AD task has also been highlighted elsewhere (Stafford et al., 2012). There are, however, a few key differences between the tasks used in the present study and those used in our previous ERP study (Bednark et al., 2013). To reduce the difficulty of the tasks, we increased the size of the “hot spot” (4.04° visual angles) and other visual stimuli (i.e., fixation cross: 0.8°; cursor: 0.8°; green sensory effect: 4.04°). Additionally, while the three different control tasks were previously conducted in two separate experiments, the same group of participants performed all control tasks in the present study. The order of the tasks was as follows: ABC-ABC-ABC-DDD or ACB-ACB-ACB-DDD. A total of 12 blocks was conducted, with a block defined by 30 presentations of the green circle. The tasks were performed on a 54 cm display with a black background. MatLab software (MathWorks, Inc.) was used for all stimulus presentation and collection of behavioral responses.

### Behavioral Analysis

For the AD task and the two action control tasks, the time it took the participants to elicit each green circle was recorded. These times were used to determine *hit rate*, or the number of green circles presented per 2 s interval. The hit rate was used as a behavioral measure of goal-directed motor performance and was calculated for the first half of green circle occurrences (1–15; F15) and second half of green circle occurrences (16–30; L15) of each block in order to investigate changes in performance within a block.

For the SR task, the time it took the participants to respond to each green circle by moving to the fixation cross, and the number of responses made were recorded. The mean response time and the mean number of responses were computed for the first half of green circle occurrences (1–15; F15) and second half of green circle occurrences (16–30; L15) of each block.

### EEG Data Acquisition and Analysis

EEG and electrooculography (EOG) data were collected continuously using a 32-channel Ag-Ag/Cl sintered Quickcap and a Neuroscan Synamps amplifier, interfaced with a Dell Intel computer running Scan 4.3 software. Data were sampled at 1000 Hz with a band pass of 0.5–200 Hz, and the gain was ×500. The 28 scalp electrode sites were referenced to linked mastoid electrodes, with AFz as the ground. Horizontal EOG data were recorded from two electrodes placed on the outer canthi of the two eyes. Vertical EOG data were recorded from linked electrodes on the infraorbital and supraorbital ridges of the left eye. Impedances were maintained below 5 kΩ.

EEG data analysis was conducted offline using purpose-written MatLab scripts. Continuous EEG data were epoched with respect sensory event onset (200 ms prior and 1000 ms after) in the behavioral task, and baseline corrected relative to the 200 ms period prior to sensory event onset. Prior to averaging, epochs containing ocular artifacts were corrected (Gratton et al., 1983). To remove movement artifact associated with PD, EEG data were then wavelet decomposed to level 9 using a “Daubechies 6” discrete wavelet transformation, and reconstructed with 1–25 Hz-frequency range. Based on visual inspection of the averaged waveforms, the mean amplitude of the fCRP for both PD and AMC groups was measured at the Cz electrode site and averaged across the time window 210–290 ms. For the novelty P3 (measured at CZ) we created a mean amplitude measure for the peak amplitude (averaged 25 ms before and after the peak) found within the 300–450 ms time window. This was done to reduce variation in the latency of the novelty P3 component observed in the patient and control group when the waveforms were visually inspected. For plotting purposes, EEG data were smoothed using a one-dimensional digital filter with a 25 ms time window.

### Statistical Analysis

The hit rate behavioral measure was entered into a mixed analysis of variance (ANOVA) with the within-subjects factors of *Task* (AD, CC, IC), *Block-Half* (F15, L15) and *Block* (Block 1, Block 2, Block 3), and between-subject factor of *Group* (patients, controls). Similar mixed ANOVAs were conducted for fCRP and novelty P3 amplitude, but the SR task added to the *Task* factor. To tease apart specific effects of interactions, additional repeated-measures ANOVAs and planned comparisons were used where appropriate to test our hypotheses. Effects were considered significant if *p* < 0.05. Greenhouse–Geisser corrections were also applied to *p*-values where appropriate. Effect sizes are shown using partial eta squared (*η*^2^). For the assessment of behavioral performance during the SR task, the mean time to response to the stimulus and the mean number of responses to the stimulus were entered into paired-sample *t*-tests that compared the PD and a control group. All statistical tests were conducted using SPSS (version 18.0) software.

## Results

### Behavioral Results

For both PD and controls, the hit rate in each task increased across blocks (*Block, F*_(2,28)_ = 18.78, *p* < 0.001, *partial η*^2^ = 0.57) and within blocks (*Block-Half, F*_(1,14)_ = 37.44, *p* < 0.001, *partial η*^2^ = 0.73). There was a significant main effect of *Task* (*F*_(2,28)_ = 72.48, *p* < 0.001, *partial η*^2^ = 0.84) demonstrating that the hit rate during the AD task (*M* = 0.85, SE = 0.08) was significantly smaller than in the CC task (*M* = 1.64, SE = 0.09, *p* < 0.001) and the IC task (*M* = 1.68, SE = 0.09, *p* < 0.001). A significant *Task × Block-Half* interaction (*F*_(2,28)_ = 33.59, *p* < 0.001, *partial η*^2^ = 0.71) suggested that performance was improving at a greater rate during the AD task. Verifying learning-related improvement, *post hoc* paired-samples *t*-tests showed that the change in hit rate *within* a block was significantly larger for the AD task (*M* = 0.40, SE = 0.05) compared to the CC task (*M* = 0.10, SE = 0.04, *t*_(47)_ = 4.69, *p* < 0.001) and the IC task (*M* = −0.06, SE = 0.05, *t*_(47)_ = 8.54, *p* < 0.001).

**Figure 2:**
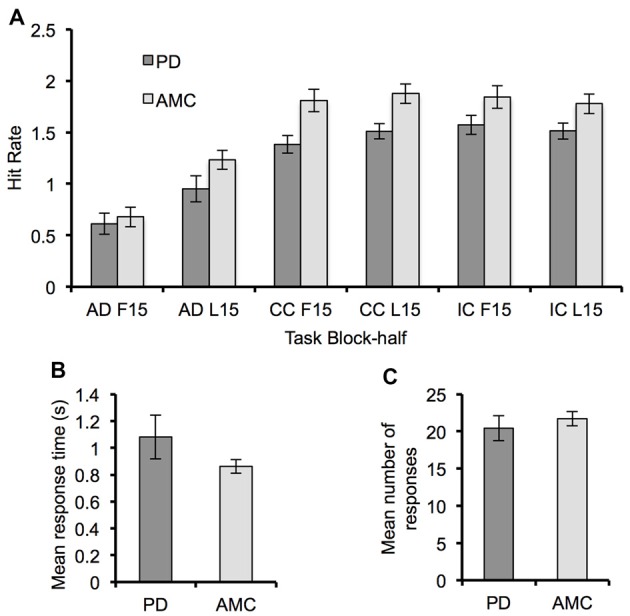
**Behavioral performance for the Parkinson’s disease (PD) and AMC groups. (A)** The hit rate for each block-half (F15, L15) of the AD task, the CC task, and the IC task, showing a general improvement in performance across all tasks. **(B)** Mean response time and **(C)** mean number of responses from the SR task for each group. Overall behavioral performance across the two groups was similar. Error bars indicate standard error.

Regarding the difference in behavioral performance between groups, we did not find any significant difference between groups in hit rate (Figure 2A). Though the hit rate of the PD group was generally less than that of the control group. There were also no significant differences between groups in mean response time (*F*_(1,14)_ = 1.69, *p* = 0.21, *partial η*^2^ = 0.11) and number of responses (*F*_(1,14)_ = 0.19, *p* = 0.67, *partial η*^2^ = 0.01) during the SR task (Figures 2B,C).

### fCRP Results

**Figure 3:**
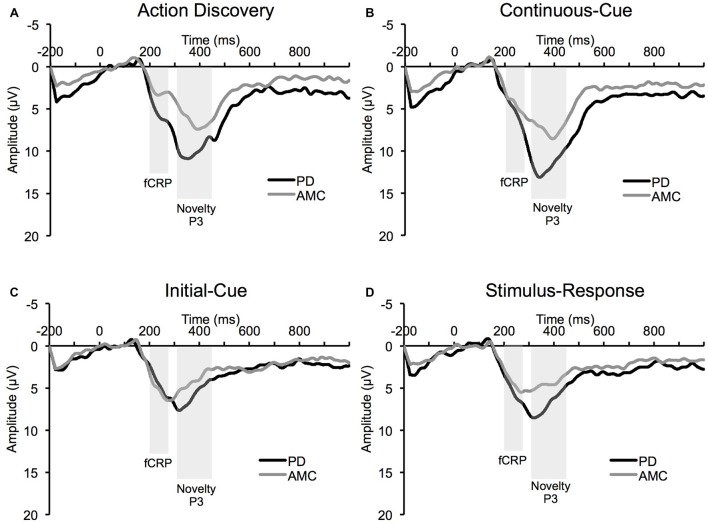
**Average event-related potential (ERP) waveform at Cz electrode site from the PD group and the AMC group during (A) the AD task, (B) the CC task, (C) the IC task, and (D) the SR task**.

**Figure 4:**
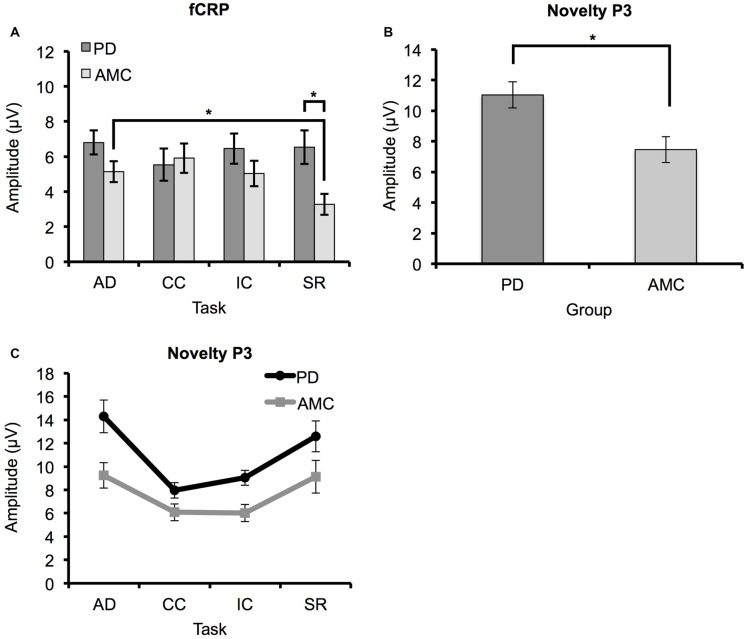
**ERP results for PD and AMC groups. (A)** Feedback correct-related positivity (fCRP) amplitude in the PD and AMC groups for the AD task, CC task, IC task, and SR task. There was a significant difference in fCRP amplitude between the PD group and the AMC group in SR. For the AMC group, fCRP amplitude in SR was significantly reduced compared to AD. **(B)** There was an overall significant enhancement in novelty P3 amplitude in the PD group compared to the AMC group. **(C)** However, the general trend of the novelty P3 was similar across the different tasks for both groups. Error bars indicate standard error. *p < 0.05.

The average ERP waveforms for each task and group are presented in Figure 3. We found a significant *Task × Group* interaction in the amplitude of the fCRP (*F*_(3,42)_ = 3.73, *p* = 0.03, *partial η*^2^ = 0.21). As shown in Figure 4A, subsequent *post hoc* analysis revealed that the magnitude of the fCRP during the SR task was significantly larger in amplitude in the PD group (*M* = 6.53 μV, SE = 0.80 μV) compared to the control group (*M* = 3.28 μV, SE = 0.80 μV; *Group, F*_(1,14)_ = 8.40, *p* = 0.012, *partial η*^2^ = 0.375; Bonferroni corrected α-level = 0.0125). Initial analysis also suggests that the magnitude of the fCRP response for both groups demonstrated a reduction across *Block-Half* (*F*_(1,14)_ = 4.84, *p* = 0.045, *partial η*^2^ = 0.26) and *Block* (*F*_(2,28)_ = 5.15, *p* = 0.013, *partial η*^2^ = 0.27). However, when each group was assessed individually, only the PD group demonstrated significant reductions in fCRP amplitude across *Block-Half* (*F*_(1,14)_ = 4.84, *p* = 0.045, *partial η*^2^ = 0.26) and *Block* (*F*_(2,28)_ = 5.15, *p* = 0.013, *partial η*^2^ = 0.27). In the control group, there was a significant main-effect of *Task* (*F*_(3,21)_ = 4.48, *p* = 0.033, *partial η*^2^ = 0.39) with the difference observed between the AD task (*M* = 5.14 μV, SE = 0.60 μV) and the SR task (*M* = 3.28 μV, SE = 0.60 μV, *p* = 0.022; Bonferroni corrected α-level = 0.016), as shown in Figure 4A.

### Novelty P3 Results

As shown in Figure 4B, the magnitude of the novelty P3 response in the PD group was significantly larger than the novelty P3 response in the control group (*Group, F*_(2,14)_ = 4.48, *p* = 0.034, *partial η*^2^ = 0.43). However, while the novelty P3 amplitude for the PD group was larger than in the control group, the general trend of novelty P3 response is similar for the two groups (Figure 4C). There was also a significant main-effect of *Task* overall, (*F*_(3,42)_ = 4.48, *p* = 0.034, *partial η*^2^ = 0.43). Bonferroni pair-wise comparisons revealed that the novelty P3 response during the AD task (*M* = 11.77 μV, SE = 0.89 μV) was significantly larger than in the IC task (*M* = 7.03 μV, SE = 0.49 μV, *p* < 0.001) and CC task (*M* = 7.53 μV, SE = 0.48 μV, *p* < 0.001). The SR task (*M* = 10.65 μV, SE = 0.96 μV) was also significantly different from the IC task (*p* = 0.003) and the CC task (*p* = 0.017). However, no difference between the AD task and the SR task was observed. The novelty P3 response also demonstrated a significant reduction across *Block-Half* (*F*_(1,14)_ = 4.48, *p* = 0.034, *partial η*^2^ = 0.43), and* Block* (*F*_(2,28)_ = 4.48, *p* = 0.034, *partial η*^2^ = 0.43).

This pattern of significant main effects was observed in both PD and control groups, as confirmed by separate analyses on each group. Accordingly, in the PD group, there were significant main effects of *Task* (*F*_(3,21)_ = 14.13, *p* = 0.001, *partial η*^2^ = 0.67), *Block-Half* (*F*_(1,7)_ = 27.27, *p* = 0.001, *partial η*^2^ = 0.80), and *Block* (*F*_(2,12)_ = 6.04, *p* = 0.021, *partial η*^2^ = 0.46). The pattern was similar for the control group: *Task* (*F*_(3,21)_ = 7.36, *p* = 0.006, *partial η*^2^ = 0.51), *Block-Half* (*F*_(1,7)_ = 13.59, *p* = 0.008, *partial η*^2^ = 0.66), and *Block* (*F*_(2,14)_ = 6.30, *p* = 0.027, *partial η*^2^ = 0.47).

## Discussion

The present study investigated the neural processes mediating the discovery of novel actions in PD patients currently receiving dopamine treatment. We found no significant behavioral differences in AD between PD patients and age-matched controls. However, ERP analysis suggests that there may be significant alterations to the neural processes involved in the formation of action-outcome associations in PD patients compared to controls.

### Novelty P3

As hypothesized, the amplitude of the novelty P3 was larger in people with PD compared to age-matched controls. This effect is complementary to previous work that has demonstrated a reduction in novelty P3 amplitude in PD patients *OFF* dopamine medication (Poceta et al., 2006). Indeed, evidence from multiple studies suggests that dopamine is a crucial neuromodulator of the novelty P3 response (Polich and Criado, 2006; Polich, 2007). This would suggest that dopamine levels modulate the response to salient unexpected sensory events, which is in keeping with the dopamine error-signaling hypothesis (Schultz, 1997, 1998). Additionally, this enhancement of the novelty P3 provides further evidence for the proposed over-abundance of dopamine in relatively preserved portions of the basal ganglia (Gotham et al., 1988; Swainson et al., 2000; Cools et al., 2001; Shohamy et al., 2006). In contrast to previous EEG studies, our results suggest that the mechanisms governing the reduction of the novelty P3 are unaffected or restored in medicated individuals with PD.

In both PD patients and age-matched controls, the novelty P3 demonstrated learning-related reductions within and across blocks of the AD task. Previously, we hypothesized that the novelty P3 reflects the engagement of attentional mechanisms necessary for identifying the movements responsible for the unexpected sensory event (Franz, 2012; Bednark et al., 2013). As the responsible movements are identified and a heuristic is formed to guide future action selection, the need to engage attentional mechanisms is reduced. In the present study, we found that the novelty P3 response in PD patients still demonstrated learning-related reductions. This would suggest that neural processes mediating action-outcome anticipation might be unaffected by changes to the magnitude of the dopamine response or that dopamine medication restores function to this system.

According to previous AD proposals of the dopamine error signal, the formation of long-term associations between actions and outcomes are likely established in structures outside the basal ganglia (Redgrave and Gurney, 2006; Redgrave et al., 2008). One such brain structure is anterior cingulate cortex (ACC). Recent evidence indicates that the ACC is responsible for governing the value of actions based on their previous reinforcement history (Walton et al., 2004; Rushworth et al., 2007; Holroyd and Coles, 2008). The ACC is the proposed source generator of the novelty P3 (Polich, 2007), and is thought to receive the dopamine error signal from the basal ganglia (Holroyd and Coles, 2002, 2008). While original proposals of this ACC-dopamine error signaling (Holroyd and Coles, 2002) have focused on prediction errors that are derived from subcortical indications of stimulus novelty (for a review see Redgrave et al., 2008, 2011), recent evidence from animal studies suggests that the dopamine error signal (with a longer latency) can be elicited from cortical indications of stimulus novelty (Bromberg-Martin et al., 2010; Nomoto et al., 2010). Along these lines, there is evidence that the ACC can directly modulate dopamine release (Gariano and Groves, 1988). Indeed, it has been proposed that different time scales of the dopamine response may control different aspects of motor behavior (Joshua et al., 2009). Thus, the ACC may govern the learning-related reductions in the novelty P3 response through its modulation of dopamine signaling. This possible top-down mechanism appears to remain moderately intact or is restored to normal function in PD patients on regular dopamine treatment.

### fCRP

In the motor tasks in which participants’ actions were responsible for the occurrence of the sensory event, there was no significant difference between PD patients and age-matched controls. Crucially, when the participants’ actions were unrelated to the occurrence of the sensory event, we observed an enhancement in the fCRP response in PD patients compared to age-matched controls. As highlighted in the Introduction, previous studies have demonstrated that the fCRP response is enhanced by the perceived responsibility over Li et al. (2011) and coupling of an intentional action with a sensory event (Bednark et al., 2013; Bednark and Franz, 2014). Additionally, it has been previously demonstrated that PD patients on regular dopamine treatment perceive actions and sensory events as occurring closer in time than when OFF dopamine treatment (Moore et al., 2010). These converging lines of evidence would suggest that PD patients had an exaggerated experience of association between their actions and the sensory effect during the SR task. This is despite explicit knowledge that their actions were not responsible for the sensory effect. Presumably this could be because dopamine treatment used to restore dopamine levels in PD patients may actually reduce the dynamic range of the dopamine response (Frank, 2005). This, in turn, may reduce the fCRP differentiation between action-related and action-unrelated sensory effects.

In contrast to the novelty P3, the pattern of fCRP response across and within tasks in PD patients varied significantly from the fCRP pattern observed in age-matched controls. This would suggest that the processes involved in the fCRP response might be altered in PD patients. A recent source localization study that used principal component analysis to remove the influence of other ERPs demonstrated that the basal ganglia is the likely source generator of the reward-related fCRP response (Foti et al., 2011). Applied to our findings, this would suggest that alterations to the fCRP response in PD patients may occur at the level of the basal ganglia. Thus, in PD patients *ON* medication, it appears that over-abundant dopamine levels in relatively preserved portions of the basal ganglia causes the behavioral relevance of the sensory effect to be maintained across all tasks.

An alternative possibility is that the enhanced fCRP may be related to increased task motivation. Others have suggested that task motivation can modulate brain activity in the time-range of the fCRP (Hajcak et al., 2005; Boksem et al., 2006; Sailer et al., 2010). However, it is unlikely that task motivation in the PD patients was only enhanced during the SR task. Nevertheless, reduced task motivation may explain the observed reduction in fCRP amplitude within and across blocks in the PD patients.

### Limitations and Future Directions

The low number of individuals with PD available for this study and the lack of an OFF-medication state in the PD group limits the extent to which these results can be generalized; the present findings must be viewed as preliminary. However, it is important to note that despite the small sample size and heterogeneity of the PD observed in these patients, we were still able to find significant differences in our ERP measures. Thus, this study provides initial evidence for the use of ERPs in exploring PD. Future studies with access to larger patient populations and *ON*/*OFF* design should be conducted to determine the extent to which these ERPs are influenced by dopamine medication during agent-based action learning. However, performing a study with *ON/OFF* design may prove to be difficult given the learning nature of this task. Differences between the *ON* and *OFF* state may not be detected because carry-over effects (e.g., learning the task) affect performance in the task from one state to the next. Conducting this experiment with de novo PD patients who still have a normal level of motor control could potentially reveal if action learning mechanisms are affected before gross motor control effects are visible.

### Conclusion

This is an initial study investigating whether the ability to identify new actions might be affected in dopamine medicated PD participants. AD is maintained in PD patients on regular dopamine treatment despite a potential over-abundance of dopamine in relatively preserved portions of the basal ganglia. This highlights the importance of structures outside the basal ganglia for the formation of long-term action-outcome representations. However, the initial experience of action-outcome association appears to be affected by increased dopamine levels in the basal ganglia.

## Author Contributions

JGB: designed and conducted study, analyzed data, and wrote the manuscript. JNJR: contributed to study design and writing of the manuscript. TS: contributed to study design, the development of the experimental task, and editing the manuscript. PR: contributed to task design and experimental concept; contributed to the editing of the manuscript. EAF: provided the facilities for conducting the study; contributed to study design, data analysis, and writing of the manuscript.

## Conflict of Interest Statement

The authors declare that the research was conducted in the absence of any commercial or financial relationships that could be construed as a potential conflict of interest.
